# A Novel Mini-DNA Barcoding Assay to Identify Processed Fins from Internationally Protected Shark Species

**DOI:** 10.1371/journal.pone.0114844

**Published:** 2015-02-03

**Authors:** Andrew T. Fields, Debra L. Abercrombie, Rowena Eng, Kevin Feldheim, Demian D. Chapman

**Affiliations:** 1 School of Marine and Atmospheric Science, Stony Brook University, Stony Brook, New York, 11794, United States of America; 2 Abercrombie & Fish, Miller Place, New York, 11764, United States of America; 3 Pritzker Laboratory for Molecular Systematics and Evolution, Field Museum of Natural History, 1400 South Lake Shore Drive, Chicago, Illinois, 60605, United States of America; Macquarie University, AUSTRALIA

## Abstract

There is a growing need to identify shark products in trade, in part due to the recent listing of five commercially important species on the Appendices of the Convention on International Trade in Endangered Species (CITES; porbeagle, *Lamna nasus*, oceanic whitetip, *Carcharhinus longimanus* scalloped hammerhead, *Sphyrna lewini*, smooth hammerhead, *S. zygaena* and great hammerhead *S. mokarran*) in addition to three species listed in the early part of this century (whale, *Rhincodon typus*, basking, *Cetorhinus maximus*, and white, *Carcharodon carcharias*). Shark fins are traded internationally to supply the Asian dried seafood market, in which they are used to make the luxury dish shark fin soup. Shark fins usually enter international trade with their skin still intact and can be identified using morphological characters or standard DNA-barcoding approaches. Once they reach Asia and are traded in this region the skin is removed and they are treated with chemicals that eliminate many key diagnostic characters and degrade their DNA (“processed fins”). Here, we present a validated mini-barcode assay based on partial sequences of the cytochrome oxidase I gene that can reliably identify the processed fins of seven of the eight CITES listed shark species. We also demonstrate that the assay can even frequently identify the species or genus of origin of shark fin soup (31 out of 50 samples).

## Introduction

The collagenous protein fibers inside shark fins, also known as the ceratotrichia, are the primary constituent of the Asian delicacy shark fin soup [1]. The trade in shark fins to supply the market for this soup is arguably the most significant driver of shark fisheries worldwide, many of which have proven to be unsustainable [2–5]. In response to this issue Parties to the Convention on International Trade in Endangered Species (CITES) have voted to list eight species of sharks on Appendix II of the Convention. These species are the whale (*Rhincodon typus*; adopted in 2001), basking (*Cetorhinus maximus*; 2001), white (*Carcharodon carcharias*; 2004), oceanic whitetip (*Carcharhinus longimanus*; 2013), scalloped hammerhead (*Sphyrna lewini*; 2013), smooth hammerhead (*S*. *zygaena*; 2013), great hammerhead (*S*. *mokarran*; 2013) and porbeagle (*Lamna nasus*; 2013). International trade of these species and their parts requires export permits certifying that the trade in each specimen is not detrimental to the survival of the species. Customs personnel of both exporting and importing nations will therefore have to be able to recognize the traded products of these species in order to identify illicit trade (i.e., trade without permits) in order to be able to effectively enforce CITES.

The initial processing of sharks fins involves sun or oven drying them after excision from the carcass. International trade from source nations to Asian markets frequently involves fins of this kind (i.e., with the skin still on). Provisional identification of these types of fins from several CITES listed species is possible using morphological characters (e.g., www.sharkfinid.org). Standard DNA barcoding (i.e., amplification of ~ 500–650 bp of the mitochondrial cytochrome *c* oxidase I gene [COI]) and polymerase chain reaction (PCR) techniques are available to then confirm species-of-origin or to identify fins that cannot be identified using morphology [6–9]. The next stage of fin processing is more intensive, however, and involves the removal of the outermost layer of skin (denticles), followed by chemical treatment to obtain the bleached, skinned retail product (hereafter referred to as a “processed fin”, [1]). Most of this processing is performed in Asia and there is significant regional international trade of these processed fins, as well some as trade outside of the region to satisfy demand for shark fin soup elsewhere [1]. The genomic DNA of these fins is degraded during processing and it is frequently difficult to obtain large PCR amplicons, potentially complicating the use of standard genetic identification techniques [6–9]. The objectives of this study were two-fold. First, we present a mini-barcode cytochrome *c* oxidase I assay that yields a short (~110–130 bp) sequence from degraded shark DNA templates (e.g., processed fins and individual ceratotrichia extracted from shark fin soup). We then show that this sequence can be used to confidently identify products from seven of the eight CITES listed shark species using freely available databases (Fish Barcode of Life Initiative [FISH-BOL] and the National Center for Biotechnology Information [NCBI] GenBank) and/or species diagnostic nucleotides.

## Methods

The mitochondrial cytochrome *c* oxidase I (COI) gene is used as a barcode for most animal life [10]. Two COI sequences from 41 shark species, including many that are common in the global fin trade [2] and all 8 of the CITES listed species were downloaded from the National Center for Biotechnology Information (NCBI) GenBank (http://www.ncbi.nlm.nih.gov/genbank/). We included at least one species from five of the eight living Orders (Carcharhiniformes, Lamniformes and Orectolobiformes, Hexanchinformes and Squaliformes). These sequences were aligned and compared with one another to identify conserved and variable regions using the sequence-editing program GeneDoc [11]. A conserved region was found ~ 150 bp upstream of the beginning of these sequences. We designed a novel reverse primer (Shark COI-MINIR 5’-AAGATTACAAAAGCGTGGGC-3’) to anneal to this conserved region. We anticipated that a multiplex polymerase chain reaction (PCR) including this primer and the two overlapping M13 labeled universal fish barcoding forward primers ([12]; FishF2_t1 5’-TGTAAAACGACGGCCAGTCGA CTAATCATAAAGATATCGG CAC-3’and VF2_t1 5’-TGTAAAACGACGGCCAGTCAACC AACCA CAAAGACATTGGC AC-3’) would yield an amplicon of ~ 150 bp with an M13 tail from all shark genomic DNA templates. This amplicon could then be sequenced using an M13 primer and would be expected in practice to yield a sequence of ~ 110–130 bp.

To validate that the assay worked as intended in terms of amplification and sequencing performance, PCR was carried out using genomic DNA template isolated from one of three types of shark tissue sample: (1) ethanol preserved tissues collected from live or dead sharks of known species identity (N = 4 individuals from each of 21 species, Table 1); (2) processed fins of unknown species origin (Table 2) sampled in retail markets (Hong Kong, N = 41) and (3) individual ceratotrichia of unknown species origin removed from shark fin soup purchased in restaurants in the U.S.A. (N = 50, Table 2). Genomic DNA was extracted from ~10–25 mg of tissue using the DNeasy commercial kit (Qiagen, Valencia, California). PCR was performed in a volume of 50 uL, which included 1 uL of the extracted genomic DNA, 10 pmol of each forward and reverse primer, 1X PCR buffer, 200 uM dNTPs, and 1 unit of HotStar© Taq Polymerase (Qiagen, Valencia, California). All reactions were run with a positive (i.e., shark genomic DNA previously confirmed to amplify with other primers) and negative (i.e., no DNA). Thermal cycling conditions consisted of a 15 min activation of the polymerase at 95°C, followed by 35 cycles of 1 min at 94°C, 1 min at 52°C, and 2 min at 72°C, followed by a final extension step of 72°C for 5 min. Amplified fragments were resolved on a 2% agarose gel and visualized using ethidium bromide dye to verify successful amplification. PCR products were purified with ExoSAP-IT (Affymetrix, Inc., Santa Clara, CA, USA) and sequenced using the Big Dye Terminator v3.1 cycle sequencing kit (Applied Biosystems, Foster City, CA, USA) with a Bio-Rad DYAD thermal cycler (Bio-Rad Laboratories, Hercules, CA, USA). The M13 forward primer was used for sequencing. The resulting products were precipitated with 125mM EDTA and 100% ethanol and run on an ABI 3730 DNA Analyzer (Applied Biosystems). Resulting sequences were validated by eye, trimmed for quality and any primer sequence present was removed.

**Table 1:** Samples of known species identity tested for amplification (AMP) and sequencing (SEQ) performance with the mini-barcode assay.

Common name	Species	N	Tests
Porbeagle	*Lamna nasus*	10	AMP/SEQ
White	*Carcharodon carcharias*	10	AMP/SEQ
Oceanic whitetip*	*Carcharhinus longimanus*	10	AMP/SEQ
Whale	*Rhincodon typus*	10	AMP/SEQ
Basking	*Cetorhinus maximus*	1	AMP/SEQ
Scalloped hammerhead*	*Sphyrna lewini*	10	AMP/SEQ
Great hammerhead*	*Sphyrna mokarran*	10	AMP/SEQ
Smooth hammerhead*	*Sphyrna zygaena*	10	AMP/SEQ
			
Bigeye thresher*	*Alopias superciliosus*	4	AMP
Common thresher*	*Alopias vulpinus*	4	AMP
Blacknose	*Carcharhinus acronotus*	4	AMP
Bignose	*Carcharhinus altimus*	4	AMP
Copper	*Carcharhinus brachyurus*	4	AMP
Spinner	*Carcharhinus brevipinna*	4	AMP
Silky*	*Carcharhinus falciformis*	4	AMP
Finetooth	*Carcharhinus isodon*	4	AMP
Bull*	*Carcharhinus leucas*	4	AMP
Blacktip	*Carcharhinus limbatus*	4	AMP
Dusky*	*Carcharhinus obscurus*	4	AMP
Caribbean reef	*Carcharhinus perezi*	4	AMP
Sandbar*	*Carcharhinus plumbeus*	4	AMP
Blackspot	*Carcharhinus sealei*	4	AMP
Night	*Carcharhinus signatus*	4	AMP
Sand tiger	*Carcharias taurus*	4	AMP
Tiger*	*Galeocerdo cuvier*	4	AMP
School	*Galeorhinus galeus*	4	AMP
Nurse	*Ginglymostoma cirratum*	4	AMP
Bigeye sixgill	*Hexanchus griseus*	4	AMP
Shortfin mako*	*Isurus oxyrinchus*	4	AMP
Longfin mako	*Isurus paucus*	4	AMP
Salmon	*Lamna ditropis*	4	AMP
Porbeagle	*Lamna nasus*	4	AMP
Sicklefin lemon	*Negaprion acutidens*	4	AMP
Lemon	*Negaprion brevirostris*	4	AMP
Broadnose sevengill	*Notorhyncus cepidianus*	4	AMP
Blue*	*Prionace glauca*	4	AMP
Milk	*Rhizoprionodon acutus*	4	AMP
Caribbean sharpnose	*Rhizoprionodon porosus*	4	AMP
Atlantic sharpnose	*Rhizoprionodon terraenovae*	4	AMP
Bonnethead	*Sphyrna tiburo*	4	AMP
Spiny dogfish	*Squalus acanthias*	4	AMP
Cuban dogfish	*Squalus cubensis*	4	AMP

* denotes a species identified by Clarke et al. (2006) as making up a large fraction of the fin trade. N = Number of individuals tested. “Tests” denotes whether the samples were used to test for positive amplification (“AMP”) and/or sequencing this amplicon (“SEQ”).

**Table 2:** Sequence matching results in NCBI BLAST and BOLD of unknown processed fins and fin soup samples.

#	Type	Loc	BOLD	BLAST top hit	Coverage	Identity	UNQ	I.D.
1	P	HK	*Carcharhinus spp.*	*Carcharhinus falciformis*	100%	100%	No	Requiem/whaler
2	P	HK	*Carcharhinus spp.*	*Carcharhinus falciformis*	100%	100%	No	Requiem/whaler
3	P	HK	*Carcharhinus spp.*	*Carcharhinus falciformis*	100%	100%	No	Requiem/whaler
4	P	HK	*Carcharhinus spp.*	*Carcharhinus falciformis*	100%	100%	No	Requiem/whaler
5	P	HK	*Carcharhinus spp.*	*Carcharhinus falciformis*	100%	100%	No	Requiem/whaler
6	P	HK	*Carcharhinus spp.*	*Carcharhinus falciformis*	100%	100%	No	Requiem/whaler
7	P	HK	*Carcharhinus spp.*	*Carcharhinus falciformis*	100%	100%	No	Requiem/whaler
8	P	HK	*Carcharhinus spp.*	*Carcharhinus falciformis*	100%	100%	No	Requiem/whaler
9	P	HK	*Carcharhinus spp.*	*Carcharhinus falciformis*	100%	100%	No	Requiem/whaler
10	P	HK	*Carcharhinus spp.*	*Carcharhinus falciformis*	100%	100%	No	Requiem/whaler
11	P	HK	*Carcharhinus spp.*	*Carcharhinus falciformis*	100%	100%	No	Requiem/whaler
12	P	HK	*Carcharhinus spp.*	*Carcharhinus falciformis*	100%	100%	No	Requiem/whaler
13	P	HK	*Carcharhinus spp.*	*Carcharhinus falciformis*	100%	100%	No	Requiem/whaler
14	P	HK	*Carcharhinus spp.*	*Carcharhinus falciformis*	100%	100%	No	Requiem/whaler
15	P	HK	*Carcharhinus spp.*	*Carcharhinus falciformis*	100%	100%	No	Requiem/whaler
16	P	HK	*Carcharhinus spp.*	*Carcharhinus falciformis*	100%	100%	No	Requiem/whaler
17	P	HK	*Carcharhinus spp.*	*Carcharhinus falciformis*	100%	100%	No	Requiem/whaler
18	P	HK	*Carcharhinus spp.*	*Carcharhinus falciformis*	100%	100%	No	Requiem/whaler
19	P	HK	*Carcharhinus spp.*	*Carcharhinus falciformis*	100%	100%	No	Requiem/whaler
20	P	HK	*Carcharhinus spp.*	*Carcharhinus falciformis*	100%	100%	No	Requiem/whaler
21	P	HK	*Carcharhinus spp.*	*Carcharhinus falciformis*	100%	100%	No	Requiem/whaler
22	P	HK	*Carcharhinus spp.*	*Carcharhinus falciformis*	100%	100%	No	Requiem/whaler
23	P	HK	*Carcharhinus spp.*	*Carcharhinus falciformis*	100%	100%	No	Requiem/whaler
24	P	HK	*Carcharhinus spp.*	*Carcharhinus falciformis*	100%	100%	No	Requiem/whaler
25	P	HK	*Carcharhinus spp.*	*Carcharhinus falciformis*	100%	100%	No	Requiem/whaler
26	P	HK	*Carcharhinus spp.*	*Carcharhinus falciformis*	100%	100%	No	Requiem/whaler
27	P	HK	*Carcharhinus spp.*	*Carcharhinus falciformis*	100%	100%	No	Requiem/whaler
28	P	HK	*Carcharhinus spp.*	*Carcharhinus falciformis*	100%	100%	No	Requiem/whaler
29	P	HK	*Carcharhinus spp.*	*Carcharhinus falciformis*	100%	100%	No	Requiem/whaler
30	P	HK	*Carcharhinus spp.*	*Carcharhinus falciformis*	100%	100%	No	Requiem/whaler
31	P	HK	*Carcharhinus spp.*	*Carcharhinus falciformis*	98%	100%	No	Requiem/whaler
32	P	HK	*Carcharhinus spp.*	*Carcharhinus falciformis*	100%	100%	No	Requiem/whaler
33	P	HK	*Carcharhinus sorrah*	*Carcharhinus sorrah*	100%	100%	Yes	Spottail
34	P	HK	*Carcharhinus spp.*	*Carcharhinus obscurus*	100%	100%	No	Requiem/whaler
35	P	HK	*Galeorhinus galeus*	*Galeorhinus galeus*	100%	100%	Yes	School
36	P	HK	*Galeorhinus galeus*	*Galeorhinus galeus*	100%	100%	Yes	School
37	P	HK	*Galeorhinus galeus*	*Galeorhinus galeus*	100%	100%	Yes	School
38	P	HK	*Galeorhinus galeus*	*Galeorhinus galeus*	100%	100%	Yes	School
39	P	HK	*Galeorhinus galeus*	*Galeorhinus galeus*	100%	100%	Yes	School
40	P	HK	*Prionace glauca*	*Prionace glauca*	100%	100%	Yes	Blue
41	P	HK	*Prionace glauca*	*Prionace glauca*	100%	100%	Yes	Blue
42	S	USA	*Prionace glauca*	*Prionace glauca*	100%	100%	Yes	Blue
43	S	USA	*Prionace glauca*	*Prionace glauca*	100%	100%	Yes	Blue
44	S	USA	*Prionace glauca*	*Prionace glauca*	100%	100%	Yes	Blue
45	S	USA	*Prionace glauca*	*Prionace glauca*	100%	100%	Yes	Blue
46	S	USA	*Prionace glauca*	*Prionace glauca*	100%	100%	Yes	Blue
47	S	USA	*Prionace glauca*	*Prionace glauca*	100%	100%	Yes	Blue
48	S	USA	*Prionace glauca*	*Prionace glauca*	100%	100%	Yes	Blue
49	S	USA	*Prionace glauca*	*Prionace glauca*	100%	100%	Yes	Blue
50	S	USA	*Prionace glauca*	*Prionace glauca*	100%	100%	Yes	Blue
51	S	USA	*Prionace glauca*	*Prionace glauca*	100%	100%	Yes	Blue
52	S	USA	*Prionace glauca*	*Prionace glauca*	100%	100%	Yes	Blue
53	S	USA	*Prionace glauca*	*Prionace glauca*	100%	100%	Yes	Blue
54	S	USA	*Prionace glauca*	*Prionace glauca*	100%	100%	Yes	Blue
55	S	USA	*Prionace glauca*	*Prionace glauca*	100%	100%	Yes	Blue
56	S	USA	*Prionace glauca*	*Prionace glauca*	100%	99%	Yes	Blue
57	S	USA	*Carcharhinus leucas*	*Carcharhinus leucas*	100%	100%	Yes	Bull
58	S	USA	*Squalus spp.*	*Squalus acanthias*	100%	100%	No	Dogfish
59	S	USA	*Mustelus schmitti*	*Mustelus schmitti*	100%	100%	Yes	Narrownose smoothound
60	S	USA	*Carcharhinus spp.*	*Carcharhinus tilstoni*	100%	100%	No	Requiem/whaler
61	S	USA	*Carcharhinus spp.*	*Carcharhinus falciformis*	100%	100%	No	Requiem/whaler
62	S	USA	*Carcharhinus spp.*	*Carcharhinus falciformis*	100%	100%	No	Requiem/whaler
63	S	USA	*Carcharhinus spp.*	*Carcharhinus amboinensis*	100%	99%	No	Requiem/whaler
64	S	USA	*Carcharhinus spp.*	*Carcharhinus amboinensis*	100%	99%	No	Requiem/whaler
65	S	USA	*Sphyrna lewini*	*Sphyrna lewini*	100%	100%	Yes	Scalloped hammerhead
66	S	USA	*Galeorhinus galeus*	*Galeorhinus galeus*	100%	100%	Yes	School
67	S	USA	*Galeorhinus galeus*	*Galeorhinus galeus*	100%	100%	Yes	School
68	S	USA	*Galeorhinus galeus*	*Galeorhinus galeus*	100%	100%	Yes	School
69	S	USA	*Galeorhinus galeus*	*Galeorhinus galeus*	100%	100%	Yes	School
70	S	USA	*Isurus oxyrinchus*	*Isurus oxyrinchus*	100%	100%	Yes	Shortfin Mako
71	S	USA	*Sphyrna zygaena*	*Sphyrna zygaena*	100%	100%	Yes	Smooth hammerhead
72	S	USA	*Sphyrna zygaena*	*Sphyrna zygaena*	99%	100%	Yes	Smooth hammerhead

# = fin sample identifier, Type = processed fin (P) or soup (S), Loc = Collection location (HK = Hong Kong, USA = United States of America), BOLD = 100% identification at the lowest taxon possible (genus or species) in a Fish Barcode of Life Initiative (FISH-BOL) search, BLAST top hit = closest match in GenBank BLAST search (Coverage, Identity and UNQ (wheter or not the match was unique to that species refer to this search), I.D. = best identification based on the two searches.

The next objective was to verify that mini-barcode sequences obtained in this manner could be used to identify CITES-listed sharks. Unknown COI sequences can be identified using searchable databases, character based approaches (i.e., diagnostic nucleotoides) or phylogenetics (e.g., Lowenstein et al. 2009). The latter approach is limited if the obtained sequences are short and was therefore not pursued in this study. We downloaded all available COI sequences for the CITES listed shark species on GenBank and the Fish Barcode of Life Initiative (FISH-BOL) online database (www.fishbol.org). Sequences were trimmed to only include the section contained within the expected fragment that we typically obtained readable sequence from. These shortened sequences were used in BOLD (FISH-BOL) and BLAST (GenBank) searches to identify them to the lowest taxon possible. We considered the species identifiable with this short COI fragment when BOLD returned a 100% species level match and when the sequence’s closest match in BLAST was unambiguously and exclusively one of the CITES listed species. We also constructed a character-based identification key for each of the CITES listed species and their closest relatives determined in a previous phylogenetic study [13–14]. The downloaded and trimmed DNA sequences from the CITES species were aligned with the same sequences from their closest relatives in BioEdit v. 7.1.3.0 [15]. A consensus sequence was created for each species and then compared to its closest relatives, with the exception of *Sphyrna tudes* because only one DNA sequence was available. For each CITES species we isolated a combination of nucleotides at multiple positions that collectively distinguish the species from its closest relatives, which we hereafter refer to as the compound character attribute or *c*CA. All unknown processed fin and soup samples were identified to the lowest taxon possible by first using the searchable BOLD and BLAST databases. If these searches indicated that they were or could possibly be one of the CITES listed species we then used the *c*CA we had previously constructed to confirm the identification.

## Results and Discussion

The mini-barcode assay we developed led to the successful amplification of the expected fragment of all of the known shark samples that we tested (Table 1). Given the taxonomic diversity of these tissue samples we suggest that it is likely that this assay will amplify all or most shark species. At this stage we can confidently state that it consistently amplifies all of the tested species, which include all sharks currently listed on CITES. The assay also amplified all 41 processed fins of unknown species identity from Hong Kong. This indicates that the degraded DNA in these types of products will typically yield sufficient PCR product for sequencing. Thirty-one out of fifty shark fin soup samples amplified and were sequenced while the remaining nineteen samples failed to amplify after multiple attempts. This demonstrates that even highly degraded, cooked shark products can in many cases be amplified and sequenced using this assay.

The partial COI sequence that would be produced by our assay was obtained from Genbank and FISH-BOL entries for white (N = 16), whale (N = 19), basking (N = 52), scalloped hammerhead (N = 215), smooth hammerhead (N = 38), great hammerhead (N = 38), oceanic whitetip (N = 34) and porbeagle sharks (N = 85). We identified several errors among the shark sequences in GenBank, as has been found for other taxa [16–19]. Two DNA sequences on GenBank identified as CITES species, for example, have DNA sequences most closely related to non-CITES species. For instance a white shark (accession number JQ654702.1) has a 99% sequence identity to blue shark *Prionace glauca* but only 83 percent sequence identity to the closest white shark. Likewise, a basking shark sequence (accession number JQ654736.1) had a 99% identity with oceanic whitetip and common thresher *Alopias vulpinus* (one or both of which were also misidentified given that they are from different orders) but only an 86% sequence identity with the most similar basking shark. After omitting these misidentified sequences each distinct partial COI haplotype (S1 Table) found for each CITES species was used as a query in BOLD and BLAST searches. All of them except the oceanic whitetip sequences were correctly identified to the species level based on this sequence alone (i.e., they were considered a 100% correct species level match based on genetic distance in BOLD and their closest BLAST match was the correct species). The tissue samples that we amplified and sequenced from these species yielded the same results (Table 1). In all searches the oceanic whitetip sequences were matched to the genus *Carcharhinus* but the mini-barcode sequences were identical or nearly identical to dusky (*Carcharhinus obscurus*) and Galapagos (*C*. *galapagensis*) sharks.

GenBank and BOLD-obtained sequences from each of the CITES listed species were aligned with its closest relatives. A *c*CA of 21 bases was constructed that was unique for each species (Table 3). All of the CITES listed species varied from its closest relatives by at least 2bp with the exception of the oceanic whitetip, which has only one base separating it from at least three other *Carcharhinus spp*. We would caution against using this base alone (at position 60, Table 3 and S1 Table) to identify this species at this stage for two reasons. First, there may be variation at this site in one or more of these species that have yet to be observed in sequenced individuals. Position 60 is also in the early part of the sequence that is not as well resolved as the rest and, consequently, we do not always use this part of the sequence to identify unknown samples (S1 Table). Overall, the expected *c*CA was present in each of the known samples that we amplified and sequenced from these species (Table 1).

**Table 3:** Compound character attributes (*c*CA) for CITES-listed shark species.

Species	57	60	66	84	85	90	91	92	97	103	111	121	126	132	138	142	147	150	157	168	174
*Carcharodon carcharias*	T	A	T	A	G	A	A	C	C	C	C	C	T	A	C	C	A	T	A	T	G
*Carcharhinus longimanus*	C	A	T	A	G	A	A	C	C	C	T	C	G	T	A	T	A	T	A	A	A
*Cetorhinus maximus*	T	A	T	A	G	G	A	C	C	C	T	T	C	C	A	C	T	T	A	T	G
*Lamna nasus*	T	A	T	A	G	A	A	C	C	C	T	C	T	K	C	C	C	C	A	T	A
*Rhincodon typus*	T	A	T	A	G	T	C	T	C	C	T	C	C	T	T	T	A	T	A	G	A
*Sphyrna lewini*	C	A	T	A	A	A	A	C	C	Y	T	C	A	A	T	T	A	T	A	A	A
*Sphyrna mokarran*	C	A	T	A	G	A	A	C	C	C	T	C	G	A	C	T	A	T	A	A	A
*Sphyrna zygaena*	C	A	T	A	G	G	A	C	C	C	T	C	R	A	T	C	A	T	A	A	A

Position numbers (bold) are from the beginning of the COI gene.

It is important to highlight that some individuals of the CITES species might have alternative polymorphisms at one or more of these proposed diagnostic sites and therefore have a slightly different *c*CA to that presented here. We therefore suggest an identification strategy that combines multiple approaches to determine whether or not a mini-barcode sequence is from a CITES listed shark species. First, the sequence should be entered in BOLD and BLAST. All but one of the CITES listed species (the oceanic whitetip) are identifiable in this manner alone but at the very least this approach can identify them to genus with 100% certainty. The species diagnosis can then be independently checked by using the *c*CA presented for each of these species in Table 3 and the alignment in S1 Table, acknowledging that slight variation from this sequence may be seen in some individuals. Oceanic whitetip mini-barcode sequences are confidently identified to genus in BOLD, while BLAST and the *c*CA narrow it down to being either an oceanic whitetip, dusky (*Carcharhinus obscurus*), Galapagos (*C*. *galapagensis*) or Caribbean reef shark (*C*. *perezi*, Table 3 and S1 Table). A fin testing like this in a CITES enforcement and monitoring context would require additional sequencing to confirm the species identity. However oceanic whitetip dorsal, pectoral and lower caudal fins have a very distinctive rounded apex even after processing that is quite unlike the more pointed fins of the dusky, Galapagos or Caribbean reef sharks [20]. This suggests that a combination of visual and mini-barcoding results enable provisional species-level identification of oceanic whitetip fins.

We used the mini-barcode assay and the proposed sample identification strategy (i.e., BOLD/BLAST search followed by visual inspection of the aligned sequence for the *c*CA) to identify processed shark products. All BOLD, BLAST and *c*CA (when needed for CITES species) results were concordant. Of 40 dried processed fins 32 were requiem sharks *Carcharhinus spp*., 5 were school sharks (*Galeorhinus galeus*), 2 were blue sharks and there was a single spot-tail (*Carcharhinus sorrah*) fin (Table 2). The *c*CA of one of the *Carcharhinus* fins was an exact match to an oceanic whitetip shark but we again caution that further sequencing should be employed to verify this. Thirty-one out of 50 shark fin soup samples amplified and were identified to genus or species (Table 2). The species found in soup were predominantly blue sharks, school sharks and requiem sharks (*Carcharhinus spp*.), but also included shortfin mako (*Isurus oxyrhyncus*), dogfish (*Squalaus spp*.), smoothound (*Mustelus schmitti*, Table 2). The BLAST/BOLD/*c*CA approach showed three soup samples originated from two CITES-listed species of hammerheads (scalloped *Sphyrna lewini* and smooth, *S*. *zygaena*, Table 2).

We envision three primary applications of this mini-DNA barcoding protocol. First, border control personnel have a legal obligation to inspect shark fin imports and exports for CITES-listed species. We suggest that our protocol could be employed to identify processed fins from these species, either by randomly sampling and genetically testing fin in trade or by testing fins that are suspected to originate from these species given their size, shape or other types of field evidence. All CITES-listed species except the oceanic whitetip are identifiable with this protocol alone. A second application stems from existing and proposed bans on trade of processed shark fins and shark fin soup in several countries and U.S. states. Our protocol may be used in law enforcement contexts to confirm that processed fin products or soup are derived from sharks as opposed to certain batoids (e.g., guitarfish), teleost fish or other substitute products that may be legally traded in these states or countries. Finally, we propose that this protocol may also be useful for basic retail fin market surveys to quantify the occurrence of different shark genera and species in this trade. The unknown samples used in this study were not collected in a systematic or random manner and thus do not provide any information on the overall species composition of the trade in our sampling regions. Nevertheless, our limited sampling efforts uncovered ongoing retail trade in the processed fins or soup derived from 2 of the CITES-listed hammerhead sharks as well as several species (e.g., soupfin, shortfin mako) that are listed as “Vulnerable” (A2bd+3d+4bd) by the International Union for the Conservation of Nature (IUCN). The mini-barcode protocol presented here could be used to monitor the contribution of certain species and/or genera to dried shark fin retail markets, which is clearly needed given the large volume of the catch [5] and the documented declines in several of the most heavily exploited or vulnerable species [3–4].

## Supporting Information

S1 Table An alignment of the consensus sequences for 31 species obtained on Genbank and BOLD together with sequences recovered from the processed fin and fin soup samples (1–72). The base numbers correspond to the base position within the COI gene with respect to the start codon. Because the *S*. *tudes* sequence is not a consensus sequence, the Genbank accession number is listed. The sample numbers correspond to those in Table 2.
(XLSX)

## References

[1] Vannuccini SF, Agriculture Organization of the United Nations (1999) Shark utilization, marketing, and trade. Rome: Food and Agriculture Organization of the United Nations

[2] Clarke SC, Magnussen JE, Abercrombie DL, McAllister MK, Shivji MS (2006) Identification of shark species composition and proportion in the Hong Kong shark fin market based on molecular genetics and trade records. Conservation Biology 20: 201–211. 16909673 10.1111/j.1523-1739.2005.00247.x

[3] Dulvy NK, Baum JK, Clarke S, Compagno LJV, Cortés E, et al (2008) You can swim but you can't hide: the global status and conservation of oceanic pelagic sharks and rays. Aquatic Conservation: Marine and Freshwater Ecosystems 18: 459–482.

[4] Ferretti F, Worm B, Britten GL, Heithaus MR, Lotze HK (2010) Patterns and ecosystem consequences of shark declines in the ocean. Ecology Letters 13: 1055–1071. 10.1111/j.1461-0248.2010.01489.x 20528897

[5] Worm B, Davis B, Kettemer L, Ward-Paige CA, Chapman D, et al (2013) Global catches, exploitation rates, and rebuilding options for sharks. Marine Policy 40: 194–204.

[6] Shivji M, Clarke S, Pank M, Natanson L, Kohler N, et al (2002) Genetic Identification of Pelagic Shark Body Parts for Conservation and Trade Monitoring. Conservation Biology 16: 1036–1047.

[7] Chapman D, Abercrombie D, Douady C, Pikitch E, Stanhopen M, et al (2003) A streamlined, bi-organelle, multiplex PCR approach to species identification: Application to global conservation and trade monitoring of the great white shark, *Carcharodon carcharias* . Conservation Genetics 4: 415–425.

[8] Abercrombie D, Clarke S, Shivji M (2005) Global-scale genetic identification of hammerhead sharks: Application to assessment of the international fin trade and law enforcement. Conservation Genetics 6: 775–788.

[9] Wong EHK, Shivji MS, Hanner RH (2009) Identifying sharks with DNA barcodes: assessing the utility of a nucleotide diagnostic approach. Molecular Ecology Resources 9: 243–256. 10.1111/j.1755-0998.2009.02653.x 21564984

[10] Dawnay N, Ogden R, McEwing R, Carvalho GR, Thorpe RS (2007) Validation of the barcoding gene COI for use in forensic genetic species identification. Forensic Science International 173: 1–6. 17300895 10.1016/j.forsciint.2006.09.013

[11] Nicholas KB, Nicholas HB (1997) GeneDoc: analysis and visualization of genetic variation. EMBNEW.NEWS 4:14 http://www.nrbsc.org/gfx/genedoc.

[12] Ward RD, Zemlak TS, Innes BH, Last PR, Hebert PDN (2005) DNA barcoding Australia's fish species. Philosophical Transactions of the Royal Society B: Biological Sciences 360: 1847–1857. 10.1098/rstb.2005.1716 PMC1609232 16214743

[13] Lowenstein JH, Amato G, Kolokotronis S-O (2009) The real maccoyii: identifying tuna sushi with DNA Barcodes—contrasting characteristic attributes and genetic distances. PLoS ONE 4: e7866 10.1371/journal.pone.0007866 19924239 PMC2773415

[14] Naylor GJP, Caira JN, Jensen K, Rosana KAM, White WT, et al. (2012) A DNA sequence–based approach to the identification of shark and ray species and its implications for global elasmobranch diversity and parasitology. Bulletin of the American Museum of Natural History: 1–26220.

[15] Hall TA (1999) BioEdit: a user-friendly biological sequence alignment editor and analysis program for Windows 95/98/NT. Nucl. Acids. Symp. Ser. 41:95–98.21.

[16] Bridge PD, Spooner BM, Roberts PJ, Panchal G (2003) On the unreliability of published DNA sequences. New Phytol. 160:43–48 10.1046/j.1469-8137.2003.00861.x 33873520

[17] Vilgalys R (2003) Taxonomic misidentifications in public DNA databases. New Phytol. 160:4–5. 10.1046/j.1469-8137.2003.00894.x 33873532

[18] Wesche PL, Gaffney DJ, Keightley PD (2004) DNA sequence error rates in GenBank records estimated using the mouse genome as a reference. DNA Seq. 15:362–364. 15621661 10.1080/10425170400008972

[19] Naylor GJP, Caira JN, Jensen K, Rosana KAM, Straube N, et al (2012) Elasmobranch Phylogeny: A Mitochondrial Estimate Based on 595 Species. In: Carrier JC, Musick JA, Heithaus MR (eds) Biology of Sharks and their Relatives, Edition 2 CRC Press, Boca Raton, Florida: 31–56 10.1007/s12262-012-0597-2

[20] Abercrombie DL, Chapman DD, Gulak SJB, Carlson JK (2013) Visual identification of fins from common elasmobranchs in the Northwest Atlantic Ocean. NMFS-SEFSC-643, 51 p.

